# Vitamin D supplementation as a control program against latent tuberculosis infection in Korean high school students

**DOI:** 10.4178/epih.e2018035

**Published:** 2018-07-27

**Authors:** Eun Hee Kim, Jong-Myon Bae

**Affiliations:** Department of Preventive Medicine, Jeju National University School of Medicine, Jeju, Korea

**Keywords:** Latent tuberculosis, Disease management, Vitamin D, Antitubercular agents, Prevention and control

## Abstract

The prevalence of latnet *Mycobacterium tuberculosis* infection (LTBI) in the first-grade high school students in South Korea was 2.1%, which was the lowest level at congregated settings in 2017. For LTBI cases refusing anti-tuberculosis (TB) medication or having poor compliance, additional support should be considered. Eight systematic reviews concluded that vitamin D (VD) deficiency is a risk factor for TB. While three of four South Korean adolescents were VD deficiency, VD supplementation could be a practical remedy to protect LTBI students of refusing anti-TB medication or having poor compliance.

## INTRODUCTION

In order to reduce the incidence of tuberculosis (TB) in the country, the Korea Centers for Disease Control and Prevention (KCDC) launched a project in 2017 to administer prophylactic anti-TB drugs to persons who had tested positive for latent *Mycobacterium tuberculosis* infection (LTBI) [1]. However, because first-grade high school students among the target groups as selected by the Korean government are not covered in the World Health Organization guidelines on the management of LTBI published in 2015 [2], it has been pointed out that there is a lack of medical basis for selection of the project target groups [3].

Nevertheless, the KCDC conducted the project in 2017 and announced its results showing that the prevalence rate of LTBI among all the subjects was 11.6%, and the prevalence of LTBI among firstgrade high school students was the lowest (2.1%) among the project target groups [4]. Although no information on how many of the LTBI-positive subjects had received anti-TB drugs is available in that article, the medication acceptance and compliance rates are expected to be low considering the public opinion regarding the adverse effects of anti-TB drugs [5]. Therefore, it can be inferred that the prophylactic effects of the LTBI screening project against the entire related project cost was the lowest for the first-grade students among the target groups. Therefore, it was concluded that there was little evidence to support a national-scale LTBI screening project for first-grade high school students in South Korea (hereafter Korea).

Meanwhile, the KCDC has no consideration on follow-up measures for LTBI-positive students who refused anti-TB medication. It should also be considered that this can cause unnecessary emotional pressure on the students and their parents. Therefore, this manuscript aimed to seek ways to prevent the progression of LTBI to active TB in LTBI-positive patients refusing to take anti-TB drugs.

## MAIN BODY

*Mycobacterium tuberculosis* (MTB), upon entry into the human body, is lysed in phagosomes inside macrophages, and if the concentration of intracellular Ca^2+^ ions does not increase, the MTB resides in the phagosomes are not lysed, thereby leading to LTBI [6]. Thus, vitamin D (VD), which is involved in calcium metabolism, is directly or indirectly involved in human immunity [7,8]. In this regard, there has been accumulating evidence that VD deficiency is a risk factor for TB infection [9,10].

Prior to the development of anti-TB drugs, cod liver oil, sunbathing, and oral/injectable VD supplementation had been used to treat active TB [11]. In addition, the epidemiological phenomena showing seasonal variation in the occurrence of TB throughout the year are interpreted to be related to VD deficiency due to the difference in the amount of sunshine [12,13].

As of the end of December 2017, eight systematic reviews regarding the association between TB and VD had been published (Table 1). The main results are summarized as follows: First, VD levels were significantly lower in patients with TB, and, thus, VD deficiency could be a risk factor for TB [14-19]. Second, the administration of VD supplements is not beneficial in the treatment of active TB [20]. Third, VD supplements are safe [21].

However, there has been no systematic review on the hypothesis that VD supplements may prevent the activation of latent TB. This is because of a lack of relevant studies. According to the results reported so far, VD levels were significantly lower in patients with LTBI than that in healthy persons [22], anti-TB immunity was strengthened in a general population who received VD, compared to those who received placebo [23], and the conversion rate of the tuberculin skin test was lower among school children taking VD, while their heights increased [24]. Based on these findings, we can hypothesize that VD may inhibit progression from LTBI to active TB [12,25].

## CONCLUSION AND SUGGESTIONS

Based on the results of the previous studies, it is concluded that VD is involved in the progression from an initial infection with TB to latent TB and active TB [8]. Therefore, it can be also inferred that the administration of VD supplements can inhibit the development of TB [14].

Meanwhile, according to data from the Korea National Health and Nutrition Examination Survey, 73.3% of Korean adolescents were in a state of VD deficiency (< 20 ng/mL) [26], and about 90% of Korean adolescents had a VD level of less than 10 ng/mL in winter and spring [27]. Therefore, the administration of VD supplements to Korean adolescents is needed for musculoskeletal metabolism as well as for boosting immunity [28].

Because VD supplements are inexpensive, it is considered, as a part of the LTBI screening project, to conduct community trials at the school level among students at high risk for LTBI to secure evidence for the efficacy of VD supplements [29]. If the budget for the related project and research is high, it may be considered to provide VD supplements to LTBI-positive students who refused anti-TB medication mainly in the winter and spring seasons when there is low sunlight. In particular, we suggest that VD be provided primarily to students with LTBI who refuse anti-TB medication. Subsequently, a randomized controlled study in the future is proposed to compare the conversion rate of active TB between those receiving VD supplements and those receiving anti-TB drugs.

## Figures and Tables

**Table 1.** Main conclusions of systematic reviews associated with VD level and activation of TB

First author (year) [Ref]	No. of selected articles	Main conclusions
Huang (2016) [14]	38	VD deficiency is a risk factor for TB
Wallis (2016) [15]	8	VD is thought to have anti-inflammatory effects
Keflie (2015) [16]	23	88.9% of TB patients had VD deficiency
Zeng (2015) [17]	15	VD level less than 25 nmol/L was significantly associated with an increased risk of TB
Sutaria (2014) [18]	7	TB patients have lower VD status; Supplementation with VD leads to improved clinical outcomes
Nnoaham (2008) [19]	7	Low serum VD levels are associated with high risk of active TB
Xia (2014) [20]	5	VD supplementation have not any beneficial effect in TB treatment
Yamshchikov (2009) [21]	13	Serious adverse events attributable to VD supplementation were rare

VD, vitamin D; TB, tuberculosis; Ref, reference number.

## SUPPLEMENTARY MATERIALS

Supplementary Material: Korean version is available at (http://www.e-epih.org/.
