# Supplementary material for: Vitamin D supplementation as a control program against latent tuberculosis infection in Korean high school students
Source: Epidemiol Health. 2018 Jul 27;40:e2018035. doi: 10.4178/epih.e2018035 (PMC6232659; doi:10.4178/epih.e2018035)
Supplement: Supplementary file 1 [file epih-40-e2018035-supplementary.pdf]

Volume: 40, Article ID: e2018035

https://doi.org/10.4178/epih.e2018035

<Perspective>

고등학생의 잠복결핵 활성화 억제를 위한 비타민 D 보충제 투여

Vitamin D supplementation as a Control Program against Latent Tuberculosis Infection in Korean High School Students

김은희[1), 배종면[1)

1) 제주대학교 의학전문대학원 예방의학교실

Eun Hee Kim[1), Jong-Myon Bae[1)

1) Department of Preventive Medicine, Jeju National University School of Medicine, Jejudo, Korea

Volume: 40, Article ID: e2018035

https://doi.org/10.4178/epih.e2018035

Abstract

The prevalence of latent tuberculosis infection (LTBI) in the first-grade high school students in Korea was 2.1%, which was the lowest level at congregated settings in 2017. For LTBI cases refusing anti-TB medication or having poor compliance, additional support should be considered. Eight systematic reviews concluded that vitamin D (VD) deficiency is a risk factor for TB. While three of four Korean adolescents were VD deficiency, VD supplementation could be a practical remedy to protect LTBI students of refusing anti-TB medication or having poor compliance.

Key Words: Latent tuberculosis, Disease Management, Vitamin D, Antitubercular agents, Prevention and control

Volume: 40, Article ID: e2018035

https://doi.org/10.4178/epih.e2018035

서론

질병관리본부가 결핵안심국가를 만들기 위하여, 잠복결핵감염 (latent mycobacterium tuberculosis infection, 이하 LTBI) 여부를 검사한 뒤 양성자에게는 항결핵제를 예방 투여한다는 사업을 2017년도부터 착수하였다 [1]. 그런데 정부가 선정한 사업 대상군 중에서 고등학교 1학년생은 2015년에 발표된 세계보건기구 (World Health Organization, WHO)의 LTBI 관리 지침 [2]에서 해당되지 않아서, 사업 대상으로의 선정에 대한 의학적 근거가 부족하다는 점이 지적되었다 [3].

그럼에도 질병관리본부는 2017년 사업을 추진하여 그 결과를 발표하였는데 [4], 전체 대상군의 LTBI 양성률 11.6%이며, 고교 1학년생은 사업 대상군들 중에서 가장 낮은 양성률 (2.1%)을 보였다. 해당 논문에서 이들 검사 양성자중 얼마가 항결핵제를 복용하였는가에 대한 정보가 없지만, 항결핵제의 부작용을 우려하는 여론 [5]을 감안하면 복용 수용률 뿐만 아니라 순응률도 낮을 것으로 예상된다. 따라서 고교 1학년생에 대한 잠복결핵 검진사업의 사업비용 대비 예방효과는 사업 대상군들 중 가장 낮다고 추론할 수 있다. 따라서 국내 고교1년생에 대한 국가 단위의 잠복결핵 검진사업을 추진할 근거가 미약하다는 결론에 도달한다.

Volume: 40, Article ID: e2018035
https://doi.org/10.4178/epih.e2018035

한편 질병관리본부는 잠복결핵 검진 양성자 중 약물복용을 거부한 학생들에 대한 사후 대응 조치에 대한 고려가 없다. 학생 당사자뿐만 아니라 학부모에게 불필요한 심적 압박을 줄 수 있다는 점도 감안해야 한다. 따라서 본 원고의 목적은 잠복결핵 양성자 중 항결핵제 복용을 거부한 대상자에게, 향후 활동성 결핵으로의 전이를 막을 수 있는 방안을 모색하는 것이다.

본론

인체 내 침입한 결핵균은 대식세포 (macrophages) 내의 포식소체 (phagosomes)에서 용해 (lysis)되는데, 만약 세포내 칼슘이온의 농도가 올라가지 않으면 포식소체 내 포획된 결핵균이 용해되지 않고 체류하게 되면서 LTBI 가 된다 [6]. 따라서 칼슘 대사에 관여하는 비타민 D 는 인체 면역에 직간접으로 관여한다 [7,8]. 이와 관련하여 비타민 D 결핍은 결핵 감염의 위험요인이란 근거들이 축적되어 왔다 [9,10].

항결핵제가 개발되기 전부터 간유 (cod liver oil), 일광욕, 경구용/주사제 비

Volume: 40, Article ID: e2018035

https://doi.org/10.4178/epih.e2018035

타민 D 투여를 활동성 결핵의 치료법으로 사용하였다 [11]. 또한, 연중 결핵 발생이 계절성 변동 (seasonal variation)을 보이는 역학적 현상도 일조량의 차이에 따른 비타민 D 결핍과 관련이 있는 것으로 해석하고 있다 [12,13]

2017년 12월 말 현재까지 결핵과 비타민 D간의 관련성에 대하 체계적 고찰 (systematic reviews)이 총 8건이 발표되었다 (Table 1). 주요 결과들을 정리하면 다음과 같다. 첫째, 결핵발생자의 비타민 D 수준이 유의하게 낮아서, 결핵 발생의 위험요소이다 [14-17,19,21]. 둘째, 비타민 D 보충제 투여는 활동성 결핵의 치료에는 도움이 되지 않는다 [18]. 셋째, 비타민 D 보충제는 안전하다 [20].

그런데 비타민 D 보충제를 복용하면 잠복결핵의 활성화를 막는다는 가설에 대한 체계적 고찰은 아직 없다. 이에 대한 연구결과들이 부족하기 때문이다. 지금까지 발표된 결과들로는 잠복결핵자의 비타민 D 수준이 건강인에 비해 유의하게 낮으며 [22], 일반인에게 비타민 D를 복용하였을 때 위약군에 비하여 항결핵 면역력이 증강되었고 [23], 비타민 D를 복용한 학동기 아동에서 tuberculin skin test 양전률이 더 낮으면서 키는 더 성장했다 [24]. 이상의 결과들에 근거할 때, LTBI에서 활동성

결핵으로의 진행을 억제한다는 가설을 제기할 수 있겠다 [12,25].

결론 및 제언

앞서의 연구결과들을 살펴보았을 때, 결핵의 초기감염이 된 이후 잠복결핵, 그리고 활동성 결핵으로 진행하는 단계에서 비타민 D가 관여한다는 결론을 도출할 수 있다 [8]. 따라서 비타민 D 보충제 투여는 결핵 발생을 억제할 수 있다는 추론 또한 가능하다 [14].

한편 국민건강영양조사에 따르면 한국 청소년의 73.3%가 비타민 D 결핍 (<20 ng/mL) 이며 [26], 겨울과 봄철에는 약 90%에서 10 ng/mL 미만인 것으로 [27] 발표되었다. 따라서 한국 청소년들에게 비타민 D 보충제 투여는 면역력 증강뿐만 아니라 근골격계 대사를 위해서라도 필요하다 [28].

비타민 D 보충제는 비용이 낮기 때문에, 잠복결핵 검진사업의 일환으로 잠복결핵 발생 고위험군에게 학교 단위의 지역시험 (community trial)을 수행하여 그

효과의 근거를 확보하는 것을 고려해 볼 수 있겠다 [29]. 만약 사업 및 연구비가 부담이 된다면, 일조량이 적은 겨울과 봄 계절에 집중적으로 비타민 D 보충제를 제공하는 것도 고려할 수 있겠다. 특히 잠복결핵 양성자 중 항결핵제 복용을 거부한 학생에게 우선적으로 비타민 D 보충제를 제공하여서, 향후 추적을 통해 활동성 결핵의 전환률에서 항결핵제 복용군과 비교하는 무작위 배정 비교임상시험을 수행할 것을 제안한다.

참고문헌

1. Centers for Disease Control and Prevention in Korea. Tuberculosis is progressing now. [cited 2017 March 25]. Available from: http://cdc.go.kr/CDC/intro/CdcKrIntro0505.jsp?menuIds=HOME001-MNU1154-MNU2557-MNU2487&fid=7947&cid=73871. (korean)

2. Getahun H, Matteelli A, Abubakar I, Aziz MA, Baddeley A, Barreira D, et al. Management of latent Mycobacterium tuberculosis infection: WHO guidelines for low tuberculosis burden countries. Eur Respir J 2015;46:1563-1576.

3. Bae JM. An opposing view on including high school students in a latent tuberculosis infection control program in Korea. Epidemiol Health 2017;39:e2017015.

4. Cho SK, Park WS, Jeong HR, Kim MJ, Park SJ, Park AY, et al. Prevalence of latent tuberculosis infection at congregated settings in the Republic of Korea, 2017. Public Health Weekly Report 2018;11(12):348-354. (korean)

5. Medical Today. Urgent suggestion for stopping the control program of latent tuberculosis infection in high school students. [cited 2017 March 25]. Available from:http://aftertherain.kr/?p=34336 (korean).

6. Trimble WS, Grinstein S. TB or not TB: calcium regulation in mycobacterial survival. Cell 2007;130(1):12-4.

7. Liu PT, Stenger S, Li H, Wenzel L, Tan BH, Krutzik SR, et al. Toll-like receptor triggering of a vitamin D-mediated human antimicrobial response. Science 2006;311(5768):1770-3.

8. Zittermann A, Pilz S, Hoffmann H, März W. Vitamin D and airway infections: a European perspective. Eur J Med Res 2016;21:14.

9. Kearns MD, Tangpricha V. The role of vitamin D in tuberculosis. J Clin Transl Endocrinol 2014;1(4):167-169.

10. Talat N, Perry S, Parsonnet J, Dawood G, Hussain R. Vitamin d deficiency and tuberculosis progression. Emerg Infect Dis 2010;16(5):853-5.

11. McCullough PJ, Lehrer DS. Vitamin D, cod liver oil, sunshine, and phototherapy: Safe, effective and forgotten tools for treating and curing tuberculosis infections - A comprehensive review. J Steroid Biochem Mol Biol 2018;177:21-29.

12. Balcells ME, García P, Tiznado C, Villarroel L, Scioscia N, Carvajal C, et al. Association of vitamin D deficiency, season of the year, and latent tuberculosis infection among household contacts. PLoS One 2017;12(4):e0175400.

13. Koh GC, Hawthorne G, Turner AM, Kunst H, Dedicoat M. Tuberculosis incidence correlates with sunshine: an ecological 28-year time series study. PLoS One 2013;8(3):e57752.

14. Huang SJ, Wang XH, Liu ZD, Cao WL, Han Y, Ma AG, et al. Vitamin D deficiency and the risk of tuberculosis: a meta-analysis. Drug Des Devel Ther 2016;11:91-102.

15. Wallis RS, Zumla A. Vitamin D as Adjunctive Host-Directed Therapy in Tuberculosis: A Systematic Review. Open Forum Infect Dis 2016;3(3):ofw151.

16. Keflie TS, Nölle N, Lambert C, Nohr D, Biesalski HK. Vitamin D deficiencies among tuberculosis patients in Africa: A systematic review. Nutrition 2015;31(10):1204-12.

17. Zeng J, Wu G, Yang W, Gu X, Liang W, Yao Y, et al. A serum vitamin D level <25nmol/l pose high tuberculosis risk: a meta-analysis. PLoS One 2015;10(5):e0126014.

18. Sutaria N, Liu CT, Chen TC. Vitamin D Status, Receptor Gene Polymorphisms, and Supplementation on Tuberculosis: A Systematic Review of Case-Control Studies and Randomized Controlled Trials. J Clin Transl Endocrinol 2014;1(4):151-160.

19. Nnoaham KE, Clarke A. Low serum vitamin D levels and tuberculosis: a systematic review and meta-analysis. Int J Epidemiol 2008;37(1):113-9.

20. Xia J, Shi L, Zhao L, Xu F. Impact of vitamin D supplementation on the outcome of tuberculosis treatment: a systematic review and meta-analysis of randomized controlled trials. Chin Med J (Engl) 2014;127(17):3127-34.

21. Yamshchikov AV, Desai NS, Blumberg HM, Ziegler TR, Tangpricha V. Vitamin D for treatment and prevention of infectious diseases: a systematic review of randomized controlled trials. Endocr Pract 2009;15(5):438-49.

22. Gibney KB, MacGregor L, Leder K, Torresi J, Marshall C, Ebeling PR, et al. Vitamin D deficiency is associated with tuberculosis and latent tuberculosis infection in immigrants from sub-Saharan Africa. Clin Infect Dis 2008;46(3):443-6.

23. Martineau AR, Wilkinson RJ, Wilkinson KA, Newton SM, Kampmann B, Hall BM, et al. A single dose of vitamin D enhances immunity to mycobacteria. Am J Respir Crit Care Med 2007;176(2):208-13.

24. Ganmaa D, Giovannucci E, Bloom BR, Fawzi W, Burr W, Batbaatar D, et al. Vitamin D, tuberculin skin test conversion, and latent tuberculosis in Mongolian school-age children: a randomized, double-blind, placebo-controlled feasibility trial. Am J Clin Nutr 2012;96(2):391-6.

25. Arnedo-Pena A, Juan-Cerdán JV, Romeu-García A, García-Ferrer D, Holguín-Gómez R, Iborra-Millet J, et al. Vitamin D status and incidence of tuberculosis

among contacts of pulmonary tuberculosis patients. Int J Tuberc Lung Dis 2015;19(1):65-9.

26. Byun EJ, Heo J, Cho SH, Lee JD, Kim HS. Suboptimal vitamin D status in Korean adolescents: a nationwide study on its prevalence, risk factors including cotinine-verified smoking status and association with atopic dermatitis and asthma. BMJ Open 2017;7(7):e016409.

27. Lee YA, Kim HY, Hong H, Kim JY, Kwon HJ, Shin CH, et al. Risk factors for low vitamin D status in Korean adolescents: the Korea National Health and Nutrition Examination Survey (KNHANES) 2008-2009. Public Health Nutr 2014;17(4):764-71.

28. Hong JY, Kim SY, Chung KS, Kim EY, Jung JY, Park MS, et al. Association between vitamin D deficiency and tuberculosis in a Korean population. Int J Tuberc Lung Dis 2014;18(1):73-8.

29. Dini C, Bianchi A. The potential role of vitamin D for prevention and treatment of tuberculosis and infectious diseases. Ann Ist Super Sanita 2012;48(3):319-27.

Volume: 40, Article ID: e2018035

https://doi.org/10.4178/epih.e2018035

Table 1. Main conclusions of systematic reviews associated with vitamin D (VD) level and activation of tuberculosis (TB)

First author (year) [Reference number]	No of selected articles	Main conclusions
**Huang (2016) [14]**	38	VD deficiency is a risk factor for TB
**Wallis (2016) [15]**	8	VD is thought to have anti-inflammatory effects.
**Keflie (2015) [16]**	23	88.9% of TB patients had VD deficiency
**Zeng (2015) [17]**	15	VD level less than 25 nmol/L was significantly associated with an increased risk of TB
**Sutaria (2014) [18]**	7	TB patients have lower VD status. Supplementation with VD leads to improved clinical outcomes
**Nnoaham (2008) [19]**	7	Low serum VD levels are associated with high risk of active TB.
**Xia (2014) [20]**	5	VD supplementation have not any beneficial effect in TB treatment
**Yamshchikov (2009) [21]**	13	Serious adverse events attributable to VD supplementation were rare.
